# Supplementary material for: Brain-inspired replay for continual learning with artificial neural networks
Source: Nat Commun. 2020 Aug 13;11:4069. doi: 10.1038/s41467-020-17866-2 (PMC7426273; doi:10.1038/s41467-020-17866-2)
Supplement: Supplementary file 1 — Supplementary Information [file 41467_2020_17866_MOESM1_ESM.pdf]

Supplementary Information

Brain-inspired replay for continual learning with artificial neural networks

van de Ven *et al*.

Supplementary Methods

Derivation of latent variable regularization term with multi-modal prior. When using conditional replay, because the prior over the latent variables $\mathbf{z}$ is no longer the standard normal distribution, the typically used closed-form expression for the latent variable regularization term $\mathcal{L}^{\text{latent}}$ (Eq. 5 in the main text) is no longer valid. Here we derive new expressions for $\mathcal{L}^{\text{latent}}$ that are valid for our Gaussian multi-modal prior with a separate mode for each class.

When the input data $\mathbf{x}$ is labelled with a hard target y (as is the case for training data from the current task), the prior distribution over the latent variables $\mathbf{z}$ reduces to the mode corresponding to class y: $p_{\boldsymbol{\chi}}(.|y)) = \mathcal{N}(\boldsymbol{\mu}^y, \boldsymbol{\sigma}^{y2}\boldsymbol{I})$. As before for the standard VAE, the posterior distribution over the latent variables $\mathbf{z}$ for an input $\mathbf{x}$ is given by $q_{\boldsymbol{\phi}}(.|\mathbf{x}) = \mathcal{N}(\boldsymbol{\mu}^{(\mathbf{x})}, {\boldsymbol{\sigma}^{(\mathbf{x})}}^2\boldsymbol{I})$. In this case we can derive a closed-form expression for $\mathcal{L}^{\text{latent}}$ as follows:

$$
\begin{aligned}
\mathcal{L}^{\text{latent}}(\mathbf{x}, y; \boldsymbol{\phi}, \boldsymbol{\chi}) &= D_{\text{KL}}(q_{\boldsymbol{\phi}}(.|\boldsymbol{x})||p_{\boldsymbol{\chi}}(.|y)) \\
&= -\int q_{\boldsymbol{\phi}}(\mathbf{z}|\mathbf{x}) \log \frac{q_{\boldsymbol{\phi}}(\mathbf{z}|\mathbf{x})}{p_{\boldsymbol{\chi}}(\mathbf{z}|y)} d\mathbf{z} \\
&= E_{\mathbf{z}\sim q_{\boldsymbol{\phi}}(.|\mathbf{x})}\left[-\log q_{\boldsymbol{\phi}}(\mathbf{z}|\mathbf{x})\right] + E_{\mathbf{z}\sim q_{\boldsymbol{\phi}}(.|\mathbf{x})}\left[\log p_{\boldsymbol{\chi}}(\mathbf{z}|y)\right] \\
&= \frac{1}{2}\sum_{j=1}^{J}\left(1 + \log({\sigma_j^{(\mathbf{x})}}^2) - \log(\sigma_j^{y2}) - \frac{\left(\mu_j^{(\mathbf{x})} - \mu_j^y\right)^2 + {\sigma_j^{(\mathbf{x})}}^2}{\sigma_j^{y2}}\right)
\end{aligned}
\qquad \textbf{(1)}
$$

whereby J is the dimensionality of the latent variables $\mathbf{z}$ (i.e., $J = 100$ for our experiments). The last equality is based on the following two simplifications:

$$
\begin{aligned}
E_{\mathbf{z}\sim q_{\boldsymbol{\phi}}(.|\mathbf{x})}\left[-\log q_{\boldsymbol{\phi}}(\mathbf{z}|\mathbf{x})\right] &= E_{\mathbf{z}\sim\mathcal{N}\left(\boldsymbol{\mu}^{(\mathbf{x})}, {\boldsymbol{\sigma}^{(\mathbf{x})}}^2\boldsymbol{I}\right)}\left[-\log\mathcal{N}\left(\mathbf{z}|\boldsymbol{\mu}^{(\mathbf{x})}, {\boldsymbol{\sigma}^{(\mathbf{x})}}^2\boldsymbol{I}\right)\right] \\
&= E_{\mathbf{z}\sim\mathcal{N}\left(\boldsymbol{\mu}^{(\mathbf{x})}, {\boldsymbol{\sigma}^{(\mathbf{x})}}^2\boldsymbol{I}\right)}\left[\frac{1}{2}(\mathbf{z} - \boldsymbol{\mu}^{(\mathbf{x})})^T\left({\boldsymbol{\sigma}^{(\mathbf{x})}}^2\boldsymbol{I}\right)^{-1}(\mathbf{z} - \boldsymbol{\mu}^{(\mathbf{x})}) + \log\left(\sqrt{(2\pi)^J|{\boldsymbol{\sigma}^{(\mathbf{x})}}^2\boldsymbol{I}|}\right)\right] \\
&= E_{\mathbf{z}\sim\mathcal{N}\left(\boldsymbol{\mu}^{(\mathbf{x})}, {\boldsymbol{\sigma}^{(\mathbf{x})}}^2\boldsymbol{I}\right)}\left[\frac{1}{2}\sum_{j=1}^{J}\frac{\left(z_j - \mu_j^{(\mathbf{x})}\right)^2}{{\sigma_j^{(\mathbf{x})}}^2}\right] + \frac{1}{2}\log\left((2\pi)^J\prod_{j=1}^{J}{\sigma_j^{(\mathbf{x})}}^2\right) \\
&= E_{\boldsymbol{\epsilon}\sim\mathcal{N}(\mathbf{0},\boldsymbol{I})}\left[\frac{1}{2}\sum_{j=1}^{J}\frac{\left(\mu_j^{(\mathbf{x})} + \sigma_j^{(\mathbf{x})}\epsilon_j - \mu_j^{(\mathbf{x})}\right)^2}{{\sigma_j^{(\mathbf{x})}}^2}\right] + \frac{J}{2}\log(2\pi) + \frac{1}{2}\sum_{j=1}^{J}\log\left({\sigma_j^{(\mathbf{x})}}^2\right) \\
&= \frac{1}{2}\sum_{j=1}^{J}E_{\epsilon\sim\mathcal{N}(0,1)}\left[\epsilon^2\right] + \frac{J}{2}\log(2\pi) + \frac{1}{2}\sum_{j=1}^{J}\log\left({\sigma_j^{(\mathbf{x})}}^2\right) \\
&= \frac{1}{2}\sum_{j=1}^{J}\left(1 + \log(2\pi) + \log\left({\sigma_j^{(\mathbf{x})}}^2\right)\right)
\end{aligned}
\qquad \textbf{(2)}
$$

$$
\begin{aligned}
E_{\mathbf{z}\sim q_{\boldsymbol{\phi}}(.|\mathbf{x})}\left[\log p_{\boldsymbol{\chi}}(\mathbf{z}|y)\right] &= E_{\mathbf{z}\sim\mathcal{N}\left(\boldsymbol{\mu}^{(\mathbf{x})},\boldsymbol{\sigma}^{(\mathbf{x})2}\boldsymbol{I}\right)}\left[\log\mathcal{N}\left(\mathbf{z}|\boldsymbol{\mu}^{y},\boldsymbol{\sigma}^{y2}\boldsymbol{I}\right)\right]\\
&= E_{\mathbf{z}\sim\mathcal{N}\left(\boldsymbol{\mu}^{(\mathbf{x})},\boldsymbol{\sigma}^{(\mathbf{x})2}\boldsymbol{I}\right)}\left[-\frac{1}{2}(\mathbf{z}-\boldsymbol{\mu}^{\boldsymbol{y}})^{T}\left(\boldsymbol{\sigma}^{y2}\boldsymbol{I}\right)^{-1}(\mathbf{z}-\boldsymbol{\mu}^{y})-\log\left(\sqrt{(2\pi)^{J}|\boldsymbol{\sigma}^{y2}\boldsymbol{I}|}\right)\right]\\
&= E_{\mathbf{z}\sim\mathcal{N}\left(\boldsymbol{\mu}^{(\mathbf{x})},\boldsymbol{\sigma}^{(\mathbf{x})2}\boldsymbol{I}\right)}\left[-\frac{1}{2}\sum_{j=1}^{J}\frac{\left(z_j-\mu_j^{y}\right)^2}{\sigma_j^{y2}}\right]-\frac{1}{2}\log\left((2\pi)^{J}\prod_{j=1}^{J}\sigma_j^{y2}\right)\\
&= E_{\boldsymbol{\epsilon}\sim\mathcal{N}(\mathbf{0},\boldsymbol{I})}\left[-\frac{1}{2}\sum_{j=1}^{J}\frac{\left(\mu_j^{(\mathbf{x})}+\sigma_j^{(\mathbf{x})}\epsilon_j-\mu_j^{y}\right)^2}{\sigma_j^{y2}}\right]-\frac{J}{2}\log(2\pi)-\frac{1}{2}\sum_{j=1}^{J}\log\left(\sigma_j^{y2}\right)\\
&= E_{\boldsymbol{\epsilon}\sim\mathcal{N}(\mathbf{0},\boldsymbol{I})}\left[-\frac{1}{2}\sum_{j=1}^{J}\frac{(\mu_j^{(\mathbf{x})}-\mu_j^{y})^2+(\mu_j^{(\mathbf{x})}-\mu_j^{y})\sigma_j^{(\mathbf{x})}\epsilon_j+(\sigma_j^{(\mathbf{x})}\epsilon_j)^2}{\sigma_j^{y2}}\right]-\frac{J}{2}\log(2\pi)-\frac{1}{2}\sum_{j=1}^{J}\log\left(\sigma_j^{y2}\right)\\
&= -\frac{1}{2}\left(\sum_{j=1}^{J}\left(\frac{\mu_j^{(\mathbf{x})}-\mu_j^{y}}{\sigma_j^{y}}\right)^2+0+\sum_{j=1}^{J}\frac{\sigma_j^{(\mathbf{x})2}}{\sigma_j^{y2}}E_{\epsilon\sim\mathcal{N}(0,1)}\left[\epsilon^2\right]+J\log(2\pi)+\sum_{j=1}^{J}\log\left(\sigma_j^{y2}\right)\right)\\
&= -\frac{1}{2}\sum_{j=1}^{J}\left(\frac{\left(\mu_j^{(\mathbf{x})}-\mu_j^{y}\right)^2+\sigma_j^{(\mathbf{x})2}}{\sigma_j^{y2}}+\log(2\pi)+\log\left(\sigma_j^{y2}\right)\right)
\end{aligned}
\tag{3}
$$

When the input data $\mathbf{x}$ is labelled with soft targets $\tilde{\mathbf{y}}$ (as is the case for replayed data), the prior distribution over the latent variables $\mathbf{z}$ is a Gaussian mixture: $p_{\boldsymbol{\chi}}(.|\tilde{\mathbf{y}}))=\sum_{c=1}^{N_{\text{classes}}}\tilde{y}_c p_{\boldsymbol{\chi}}(.|c)=\sum_{c=1}^{N_{\text{classes}}}\tilde{y}_c\mathcal{N}\left(\boldsymbol{\mu}^{c},\boldsymbol{\sigma}^{c2}\boldsymbol{I}\right)$. Now it is no longer possible to find a closed-form expression for $\mathcal{L}^{\text{latent}}$. Instead we resort to estimation by sampling, for which it is useful to express $\mathcal{L}^{\text{latent}}$ as follows :

$$
\begin{aligned}
\mathcal{L}^{\text{latent}}(\mathbf{x},\tilde{\mathbf{y}};\boldsymbol{\phi},\boldsymbol{\chi}) &= D_{\text{KL}}(q_{\boldsymbol{\phi}}(.|\mathbf{x})||p_{\boldsymbol{\chi}}(.|\tilde{\mathbf{y}}))\\
&= -\int q_{\boldsymbol{\phi}}(\mathbf{z}|\mathbf{x})\log\frac{q_{\boldsymbol{\phi}}(\mathbf{z}|\mathbf{x})}{p_{\boldsymbol{\chi}}(\mathbf{z}|\tilde{\mathbf{y}})}d\mathbf{z}\\
&= E_{\mathbf{z}\sim q_{\boldsymbol{\phi}}(.|\mathbf{x})}\left[-\log q_{\boldsymbol{\phi}}(\mathbf{z}|\mathbf{x})\right]+E_{\mathbf{z}\sim q_{\boldsymbol{\phi}}(.|\mathbf{x})}\left[\log p_{\boldsymbol{\chi}}(\mathbf{z}|\tilde{\mathbf{y}})\right]\\
&= \frac{1}{2}\sum_{j=1}^{J}\left(1+\log(2\pi)+\log\left(\sigma_j^{(\mathbf{x})2}\right)\right)+E_{\boldsymbol{\epsilon}\sim\mathcal{N}(\mathbf{0},\boldsymbol{I})}\left[\log\left(\sum_{c=1}^{N_{\text{classes}}}\tilde{y}_c\mathcal{N}\left(\boldsymbol{\mu}^{(\mathbf{x})}+\boldsymbol{\sigma}^{(\mathbf{x})}\odot\boldsymbol{\epsilon}|\boldsymbol{\mu}^{c},\boldsymbol{\sigma}^{c2}\boldsymbol{I}\right)\right)\right]
\end{aligned}
\tag{4}
$$

whereby the last equality uses Eq. 2 and the reparameterization $\mathbf{z}=\boldsymbol{\mu}^{(\mathbf{x})}+\boldsymbol{\sigma}^{(\mathbf{x})}\odot\boldsymbol{\epsilon}$. Because of this reparameterization, the Monte Carlo estimate of the final expectation in Eq. 4 is differentiable with respect to $\boldsymbol{\phi}$.

Traditional measures for evaluating VAE performance. In addition to the measures for evaluating generator performance described and reported in the main text (Fig. 9), here we report the average estimated log-likelihood (Supplementary Fig. 4A) and the reconstruction error (Supplementary Fig. 4B). Similar as in the main text, these measures are compared for different variants of generative replay in the Class-IL scenario of split CIFAR-100. However, we note that these measures have several issues associated with them (1, 2) and that using them to compare between VAE models at the internal level versus at the pixel level might not be fair. Both measures were computed after the model was incrementally trained on all 100 classes and they were averaged over all $10,000$ samples in the test set.

Because we used a VAE with a deterministic decoder (see Methods in the main text), it was not possible to directly estimate a sample's likelihood using methods based on Monte Carlo sampling. As a workaround, we assumed a Gaussian observation model with unit variance (i.e., samples returned by the decoder were convolved with a multivariate Gaussian, see also 3). This way we estimated the log-likelihood of every sample in the test set using $S=100$ importance samples. For models replaying at the pixel level, the estimated log-likelihood of a sample $\mathbf{x}$ was given by:

$$
\text{ll}_{\text{pixel}}(\mathbf{x})=\log\frac{1}{S}\sum_{s=1}^{S}\frac{p_{\text{obs}}\left(\mathbf{x}|\hat{\mathbf{x}}^{(\mathbf{x},s)}\right)p\left(\mathbf{z}^{(\mathbf{x},s)}\right)}{q_{\boldsymbol{\phi}}\left(\mathbf{z}^{(\mathbf{x},s)}|\mathbf{x}\right)}
\tag{5}
$$

whereby $p_{\text{obs}}(.|\hat{\mathbf{x}}) = \mathcal{N}(\hat{\mathbf{x}}, \boldsymbol{I})$ is the pixel-level likelihood defined by the Gaussian observation model given reconstructed image $\hat{\mathbf{x}}$, $p(.)$ is the prior distribution over the latent variables $\mathbf{z}$ (i.e., either $\mathcal{N}\left(\boldsymbol{\mu}^y, \boldsymbol{\sigma}^{y2}\boldsymbol{I}\right)$ for conditional replay or $\mathcal{N}(\mathbf{0}, \boldsymbol{I})$ otherwise), $q_{\boldsymbol{\phi}}(.|\mathbf{x}) = \mathcal{N}\left(\boldsymbol{\mu}^{(\mathbf{x})}, {\boldsymbol{\sigma}^{(\mathbf{x})}}^2\boldsymbol{I}\right)$ is the posterior distribution over the latent variables $\mathbf{z}$ for input $\mathbf{x}$ with $\boldsymbol{\mu}^{(\mathbf{x})}$ and $\boldsymbol{\sigma}^{(\mathbf{x})}$ the outputs of the encoder network, $\mathbf{z}^{(\mathbf{x},s)}$ is the sth importance sample from $q_{\boldsymbol{\phi}}(.|\mathbf{x})$ and $\hat{\mathbf{x}}^{(\mathbf{x},s)}$ is the reconstructed image obtained by putting $\mathbf{z}^{(\mathbf{x},s)}$ through the decoder network. For models replaying at the internal level, the estimated log-likelihood of a sample $\mathbf{x}$ was given by:

$$\text{ll}_{\text{internal}}(\mathbf{x}) = \log \frac{1}{S} \sum_{s=1}^{S} \frac{p^*_{\text{obs}}\left(\mathbf{h}^{(\mathbf{x})}|\hat{\mathbf{h}}^{(\mathbf{x},s)}\right) p\left(\mathbf{z}^{(\mathbf{x},s)}\right)}{q_{\boldsymbol{\phi}}\left(\mathbf{z}^{(\mathbf{x},s)}|\mathbf{x}\right)} \quad \textbf{(6)}$$

whereby $p^*_{\text{obs}}(.|\hat{\mathbf{h}}) = \mathcal{N}(\hat{\mathbf{h}}, \boldsymbol{I})$ is the internal-level likelihood defined by the Gaussian observation model given reconstructed internal activation vector $\hat{\mathbf{h}}$, $\mathbf{h}^{(\mathbf{x})}$ is the vector of internal activations obtained by putting the input image $\mathbf{x}$ through the convolutional layers and $\hat{\mathbf{h}}^{(\mathbf{x},s)}$ is the reconstructed internal activation vector obtained by putting $\mathbf{z}^{(\mathbf{x},s)}$ through the decoder network without deconvolutional layers.

The reconstruction error was calculated as the mean-squared error between the original image or internal activation vector and its reconstructed equivalent. To obtain these reconstructions, instead of a sample from the distribution parameterized by the output of the encoder (i.e., $q_{\boldsymbol{\phi}}(.|\mathbf{x}) = \mathcal{N}\left(\boldsymbol{\mu}^{(\mathbf{x})}, {\boldsymbol{\sigma}^{(\mathbf{x})}}^2\boldsymbol{I}\right)$), the mean $\boldsymbol{\mu}^{(\mathbf{x})}$ of that distribution was inputted into the decoder network. The reconstruction error at the pixel level was given by:

$$\text{MSE}_{\text{pixel}}(\mathbf{x}) = \frac{1}{N_{\text{pixels}}} \sum_{p=1}^{N_{\text{pixels}}} \left(x_p - \hat{x}_p^{(\mathbf{x},\text{-})}\right)^2 \quad \textbf{(7)}$$

whereby x_p is the pth pixel of the original image $\mathbf{x}$ and $\hat{x}_p^{(\mathbf{x},\text{-})}$ is the pth pixel of $\hat{\mathbf{x}}^{(\mathbf{x},\text{-})}$, which is the reconstructed image obtained by putting $\boldsymbol{\mu}^{(\mathbf{x})}$ through the decoder network. The reconstruction error at the internal level was given by:

$$\text{MSE}_{\text{internal}}(\mathbf{x}) = \frac{1}{N_{\text{units}}} \sum_{i=1}^{N_{\text{units}}} \left(h_i^{(\mathbf{x})} - \hat{h}_i^{(\mathbf{x},\text{-})}\right)^2 \quad \textbf{(8)}$$

whereby $h_i^{(\mathbf{x})}$ is the ith element of internal activation vector $\mathbf{h}^{(\mathbf{x})}$ obtained by putting the original image $\mathbf{x}$ through the convolutional layers and $\hat{h}_i^{(\mathbf{x},\text{-})}$ is the ith element of $\hat{\mathbf{h}}^{(\mathbf{x},\text{-})}$, which is the reconstructed vector of internal activations obtained by putting $\boldsymbol{\mu}^{(\mathbf{x})}$ through the decoder network without deconvoluational layers.

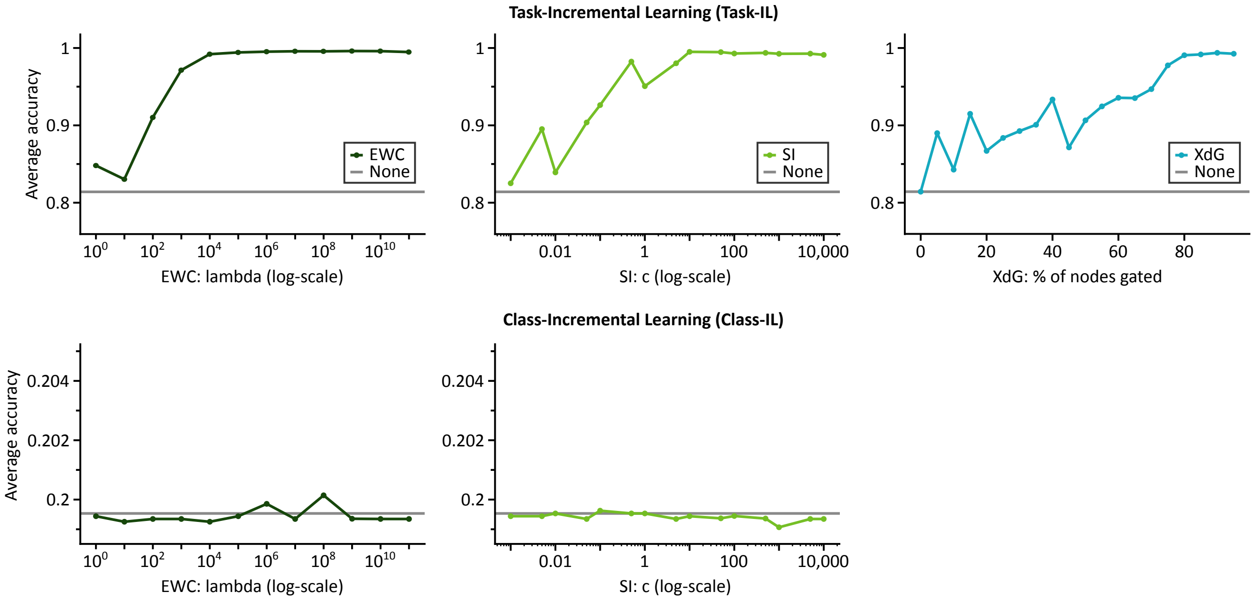

Supplementary Fig. 1 Grid searches for split MNIST. Shown are the final average test set accuracies (over all 5 tasks / based on all 10 digits) for the hyperparameter-values tested for each method. For these grid searches each experiment was run once, after which 20 new runs with different random seeds were executed using the selected hyperparameter-values to obtain the results reported in the main text. EWC: elastic weight consolidation, SI: synaptic intelligence, XdG: context-dependent gating, None: sequential training in standard way.

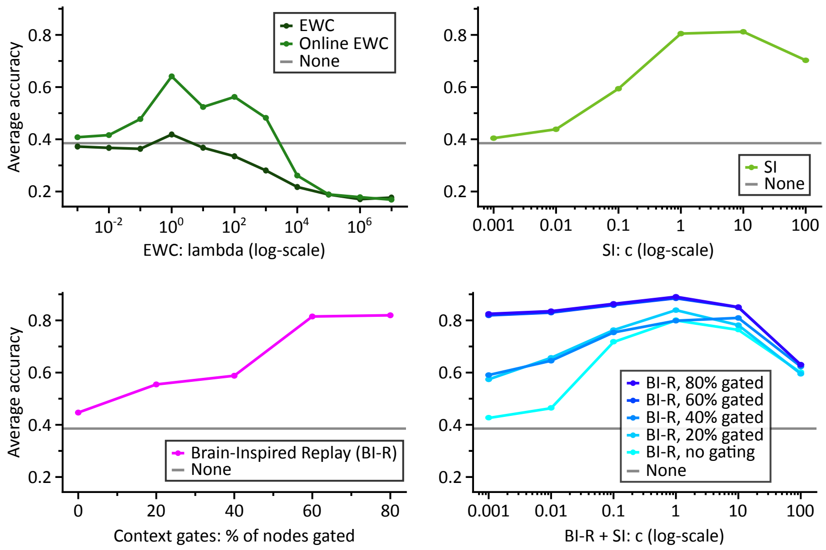

Supplementary Fig. 2 Grid searches for permuted MNIST. Shown are the final average test set accuracies (over all 100 tasks) for the hyperparameter-values tested for each method. For these grid searches each experiment was run once, after which 5 new runs with different random seeds were executed using the selected hyperparameter-values to obtain the results reported in the main text. EWC: elastic weight consolidation, SI: synaptic intelligence, BI-R: brain-inspired replay, None: sequential training in standard way.

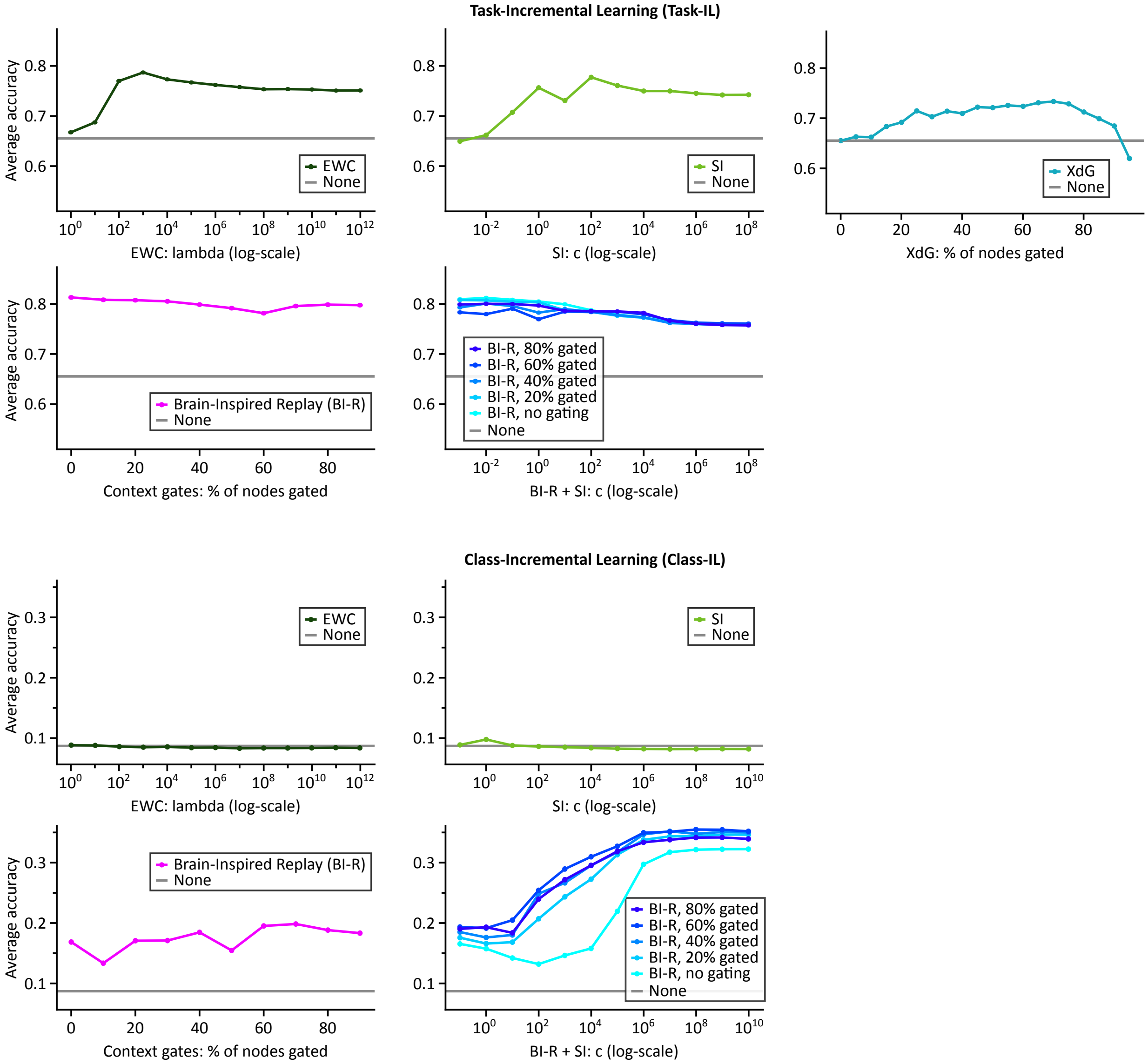

Supplementary Fig. 3 Grid searches for split CIFAR-100. Shown are the final average test set accuracies (over all 10 tasks / based on all 100 classes) for the hyperparameter-values tested for each method. Note that for the task-incremental learning scenario, combining brain-inspired replay (BI-R) with synaptic intelligence (SI) did not result in an improvement, which is why for this scenario the performance of this combination is not reported in the main text. For these grid searches each experiment was run once, after which 10 new runs with different random seeds were executed using the selected hyperparameter-values to obtain the results reported in the main text. EWC: elastic weight consolidation, XdG: context-dependent gating, None: sequential training in standard way.

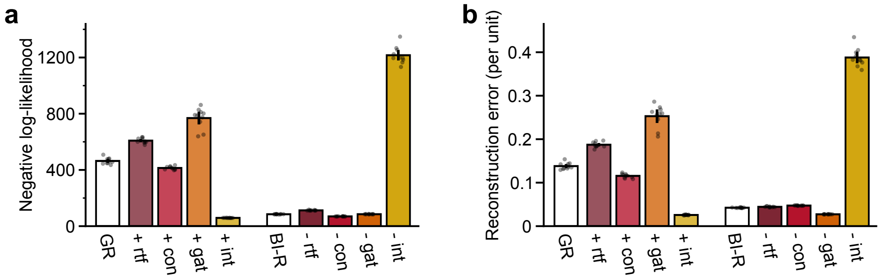

Supplementary Fig. 4 Traditional measures for evaluating VAE performance. For the class-incremental learning scenario on CIFAR-100, compared are the performance of the VAE generator for standard generative replay (GR) with individual modifications added ('+', left within each panel) and for brain-inspired replay (BI-R) with individual modifications removed ('-', right). All measures are computed on a held-out test set after each model was incrementally trained on all 100 classes. **a** Average estimated negative log-likelihood. Lower means better. **b** Reconstruction error. Lower means better. Each bar reflects the mean over 10 repetitions, error bars are ± 1 SEM, individual repetitions are indicated by dots. rtf: replay-through-feedback, con: conditional replay, gat: gating based on internal context, int: internal replay, dis: distillation.

Supplementary References

1. Theis, L., Oord, A. v. d. & Bethge, M. A note on the evaluation of generative models. Preprint at https://arxiv.org/abs/1511.01844 (2015).
2. Borji, A. Pros and cons of gan evaluation measures. *Comput. Vis. Image Underst.* **179**, 41–65 (2019).
3. Wu, Y., Burda, Y., Salakhutdinov, R. & Grosse, R. On the quantitative analysis of decoder-based generative models. In *International Conference on Learning Representations* (2017).
